# Applications of Lab on a Chip in Antimicrobial Susceptibility of *Staphylococcus aureus*: A Systematic Review

**DOI:** 10.3390/medicina59101719

**Published:** 2023-09-26

**Authors:** Carlos M. Ardila, Mateo Zuluaga-Gómez, Annie Marcela Vivares-Builes

**Affiliations:** 1 Basic Studies Department, School of Dentistry, Universidad de Antioquia UdeA, Medellín 050010, Colombia; 2 Emergency Department, Universidad Pontificia Bolivariana, Medellín 050010, Colombia; mateo.zuluaga@sanvicentefundacion.com; 3 Hospital San Vicente Fundación, Rionegro 054047, Colombia; 4 School of Dentistry, Institución Universitaria Visión de Las Américas, Medellín 050031, Colombia; anny.vivares@uam.edu.co

**Keywords:** lab on a chip device, microchip, microfluidics, *Staphylococcus aureus*, microbial sensitivity tests, minimum inhibitory concentration

## Abstract

*Background and Objectives*: *Staphylococcus aureus* is a prevalent bacterium capable of inducing various infections, including skin and soft tissue infections, bloodstream infections, pneumonia, and surgical site infections. The emergence of antimicrobial resistance in *S. aureus*, particularly methicillin-resistant *S. aureus*, has raised substantial concerns within global healthcare settings. Prior to antibiotic prescription, the ideal approach is antimicrobial susceptibility testing (AST); however, this is frequently perceived as excessively complex and time-intensive. Lab-on-a-chip (LOC) technology holds promise in addressing these challenges and advancing fundamental microbiological research while also aiding in the development of therapeutic strategies. This systematic review aims to evaluate the potential utility of LOC for AST of *S. aureus*. *Materials and Methods*: This study adhered to the PRISMA guidelines. Various databases, including SCOPUS, PubMed/MEDLINE, SCIELO, and LILACS, in addition to gray literature sources, were employed in the review process. *Results*: Sixteen studies were included in this systematic review. All these studies detailed the effectiveness, rapidity, and predictability of LOC systems for assessing *S. aureus* susceptibility to various antibiotics. When comparing the LOC approach to traditional manual methods, it was evident that LOC requires a minimal quantity of reagents. Furthermore, most studies reported that the entire LOC procedure took 10 min to 7 h, with results being equally accurate as those obtained through traditional AST protocols. *Conclusions*: The potential application of LOC for AST of *S. aureus* is emphasized by its ability to provide rapid access to minimum inhibitory concentration data, which can substantially aid in selecting the most suitable antibiotics and dosages for treating challenging infections caused by this microorganism. Moreover, the rapid AST facilitated by LOC holds promise for enhancing the appropriateness and efficacy of therapy in clinical settings.

## 1. Introduction

The major threat posed by antimicrobial resistance to the world’s healthcare systems makes it difficult for modern medicine to control infectious diseases effectively. Infections with antimicrobial resistance cause 33,000 fatalities annually in the European Union and about 35,000 deaths annually in the USA [1,2,3]. Most bacterial-related deaths could be treated with antibiotics, but according to the World Health Organization, the number of deaths brought on by bacteria resistant to antimicrobials is expected to rise to almost 10 million per year by 2050 [4,5].

*Staphylococcus aureus* is a Gram-positive, human commensal bacterium that is regularly found on healthy people’s skin. *S. aureus* can cause a range of infections, including skin and soft tissue infections, bloodstream infections, pneumonia, and surgical site infections. It is a major pathogen that may originate bacteremia as well as more serious and difficult-to-treat osteoarticular infections and heart infections such as infective endocarditis [5,6,7]. Antimicrobial resistance in *S. aureus*, particularly methicillin-resistant *S. aureus* (MRSA), has been a significant concern in healthcare settings worldwide. MRSA infections can be challenging to treat as they are resistant to commonly used antibiotics like methicillin and other beta-lactam drugs [6,7].

The impact of deaths specifically attributed to antimicrobial resistance in *S. aureus* can vary depending on several factors, such as geographic location, healthcare settings, population demographics, and access to appropriate healthcare. It is difficult to provide precise global figures on deaths solely caused by antimicrobial resistance in *S. aureus*, as often these infections occur in conjunction with other underlying health conditions [5,6,7]. However, it is well-documented that MRSA infections can result in increased morbidity, mortality, and healthcare costs compared to infections caused by non-resistant strains of *S. aureus*. According to estimates from the Centers for Disease Control and Prevention (CDC) in the United States, MRSA infections were associated with approximately 11,000 deaths in 2017. These numbers include both healthcare-associated and community-associated MRSA infections [8]. To combat the impact of antimicrobial resistance in *S. aureus* and reduce associated deaths, healthcare facilities have implemented strategies such as improved infection control practices, screening and isolation of patients carrying MRSA, and appropriate antibiotic prescribing guidelines [1,2,4,6,8]. 

The vital first step in providing patients with appropriate care is the prompt and correct identification of the causal agent responsible for infection [9,10]. This initial stage directs patient treatment plans and the efficient use of antibiotics for bacterial infections. To reduce the spread of antibiotic resistance, proper medicine use is essential [11,12]. Studying certain bacterial phenotypes requires an understanding of how bacteria interact with cells in physiological settings. The minimum inhibitory concentration (MIC) of antibiotics, which defines the likelihood of a specific drug’s therapeutic efficacy against a given infection, is determined using antibiotic susceptibility testing. Conducting bacterial categorization studies is also helpful in many applications as complementary elements to support treatment decision-making. The collection of results from antibiotic susceptibility testing and bacterial classification can take up to 48 h or longer, which can cause delays in the prescription of appropriate antibiotics or the administration of inappropriate antibiotics before obtaining antibiotic susceptibility results, impacting morbidity, mortality, the gravity of infections, and the incidence of antibiotic resistance [13,14,15].

Microscopy, cell infection models, and more contemporary molecular, cellular, and immunological assays are examples of traditional microbiological in vitro techniques [13]. These techniques have given and will continue to give invaluable information that will help us understand the host-bacterial interaction’s molecular and cellular microbiology. Nevertheless, traditional methods can have certain drawbacks [14]. Microorganisms that cannot be cultivated in vitro still exist despite advancements in culture techniques. Ex vivo and in vitro models, on the other hand, are unable to accurately replicate the physiological environment [14,15]. As a result, outcomes obtained in vitro and in vivo are not necessarily comparable. Moreover, some phenotypes, like the formation of biofilms, are conventionally researched in simple settings that do not accurately reflect complicated physiological circumstances [14,16]. 

The recent development of lab on a chip, however, can help to address some of these issues and enhance fundamental microbiological research while also assisting in the creation of therapeutic strategies.

A lab on a chip refers to a miniaturized device that integrates multiple laboratory functions onto a single microchip or small platform. It aims to replicate and perform various laboratory processes and analyses in a portable and efficient manner. Lab-on-a-chip technology has gained significant attention in recent years due to its potential applications in healthcare diagnostics, environmental monitoring, and biological research [14,17]. The concept behind lab on a chip is to downscale and miniaturize laboratory processes, such as sample preparation, mixing, separation, detection, and analysis, onto a small chip or platform. This integration of multiple functions onto a single device offers several advantages, including reduced sample and reagent volumes, faster analysis times, improved precision, and increased automation [14,18].

Lab-on-a-chip devices typically consist of microfluidic channels, chambers, valves, sensors, and detection systems, all fabricated on a small chip using techniques such as lithography, etching, and bonding. These microfluidic channels allow precise manipulation and control of fluids, enabling the handling and processing of small volumes of samples and reagents [14,17,18].

Recently, the use of microfluidics for assessing antibiotic susceptibility has gained popularity. These devices provide quick, high-throughput, and inexpensive studies using fluidic channels with a diameter of a few micrometers [19]. A droplet-based microfluidic system that confines single bacteria and medications into plugs of small volume for single-drug antimicrobial susceptibility tests has been reported to shorten detection times [20]. An alternative method for quickly isolating microorganisms is to use an inertial microfluidic chip to perform an antibiotic susceptibility test followed by a hybridization-based RNA detection method [21]. Additionally, molecular diffusion has been used to detect microbial growth on the surface of numerous gradient zones created in hydrogels by drugs or drug combinations, and MIC data were achieved after 3 h of incubation [22]. It is important to note that prior to prescribing antibiotics, antimicrobial susceptibility testing is ideal but is typically viewed as being excessively complicated and time-consuming. 

Considering what was previously described, this systematic review aims to assess the potential application of lab on a chip for antibiotic susceptibility testing of *S. aureus.*

## 2. Materials and Methods

### 2.1. Search Approach

This study’s analysis followed PRISMA (Preferred Reporting Items for Systematic Reviews and Meta-analyses) guidelines [23]. Several databases, including SCOPUS, PubMed/MEDLINE, SCIELO, and LILACS, as well as the gray literature, were used in the review framework. Up to April 2023, searches using keywords and MeSH terms included the terms organ on a chip, lab on a chip, microphysiological systems, microfluidics, bioassays, minimal inhibitory concentration, antimicrobial resistance, antimicrobial susceptibility test, biofilms, *Staphylococcus aureus*, and studies published in all languages. The next exploration included searching databases using Boolean operators (AND, OR): “organ on a chip” OR “lab on a chip” OR “microphysiological systems” OR “microfluidics” OR “bioassays” OR “minimal inhibitory concentration” OR “antimicrobial resistance” OR “antimicrobial susceptibility test” OR “biofilms” AND “*Staphylococcus aureus*”.

### 2.2. Selection Criteria

The studies that were taken into consideration for this evaluation must use microfluidic platforms or lab-on-a-chip devices and 3D printing and/or bioprinting methods to organ-on-a-chip technology.

Abstracts, reviews, systematic reviews, meta-analyses, brief communications, conference articles, patents, case reports, and studies lacking critical information on the manufacturing process were also disregarded.

### 2.3. Question

This comprehensive review aims to answer the question, what potential clinical applicability of lab on a chip for antibiotic susceptibility testing exists in experiments with *S. aureus*? 

P: experimentation with *S. aureus*

I: lab on a chip

C: control experiments

O: potential clinical applicability for antibiotic susceptibility testing

### 2.4. Review Course

To identify publications that might be suitable, two researchers examined the titles and abstracts. Given the probability of divergence in the choice of studies, a third author (MZ) could mediate. The statistical test Kappa was used to determine the importance of observer agreement (>90).

### 2.5. Data Compilation

The most significant information from the chosen studies was compiled into a table. Each researcher completed this method individually. After that, the data were compared. The data included the names of the authors, the publication date, the application of the lab-on-a-chip device, and information about its key characteristics, such as the materials used in building it, culture, bacterial strains and growth conditions, MICs, antibiotics utilized, and the main findings.

### 2.6. Risk of Bias

Two authors evaluated the quality and potential for bias in the included studies using a scale that had previously been published [24]. With the help of the tool, it is possible to assess the study using 15 different criteria, including the following: design (objective, sample, baseline characteristics, co-interventions), measures studied (measurement method, blinding (examiner, statistician), described reliability, level of agreement), statistical analysis (appropriate analysis, co-interventions, subgroup analysis, statistical significance, confidence intervals), and clinical significance.

## 3. Results

**Figure 1:**
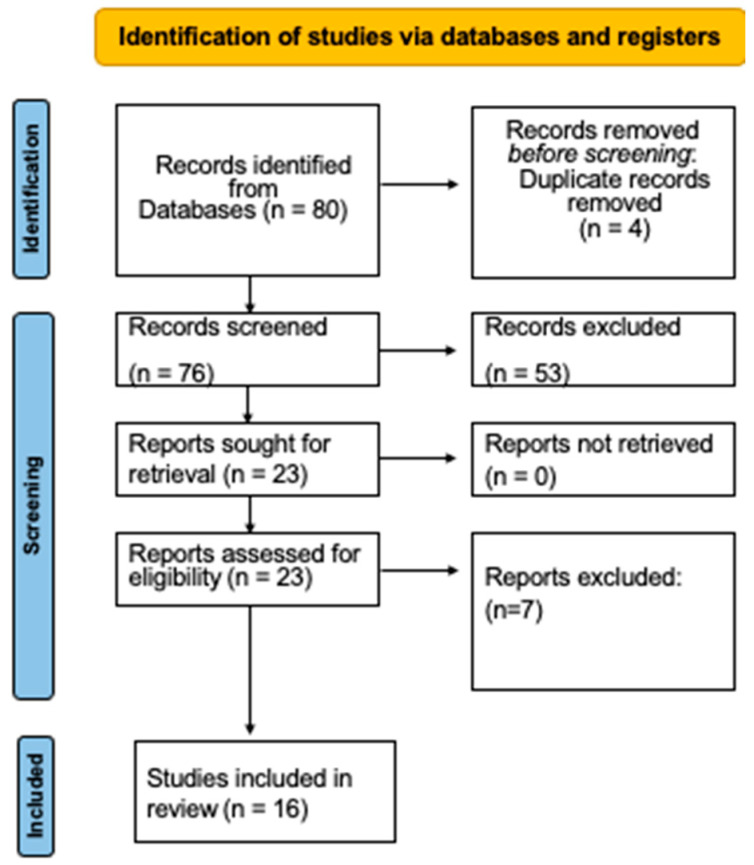
Diagram of the selection process.

A total of 80 articles were identified after the initial search. Then, 53 publications were excluded (because they did not study *S. aureus* or, because they were aimed only at the identification of the microorganism, or they investigated genes or proteins and did not assess antimicrobial susceptibility). Four papers that appeared twice were not included. After reading the entire text, seven other studies were excluded since they did not meet the selection criteria. Finally, 16 studies [19,20,25,26,27,28,29,30,31,32,33,34,35,36,37,38] were included in this systematic review (Figure 1).

The features of the included studies are shown in Table 1. Between 2008 [20] and 2021 [25], the studies included were published.

As observed in Table 1, the studies evaluated different lab-on-a-chip technologies to mainly establish the susceptibility of *S. aureus* to different antibiotics. All studies described effective, rapid, and predictable systems to assess the susceptibility of *S. aureus* to different antibiotics. Comparing the lab-on-a-chip approach to the traditional manual methods, it was found that lab-on-a-chip needs a very small number of reagents. Besides, most studies reported that the entire antimicrobial susceptibility test procedure lasted from 10 min to 7 h, and the results are equally accurate as the results of the Clinical & Laboratory Standards Institute (CLSI) [39] and the European Committee on Antimicrobial Susceptibility Testing (EUCAST, Europe) [40]. It was also observed that lab on a chip has the potential to identify *S. aureus* with high sensitivity and speed in as little as 10 min by forming and measuring small volume plugs and is less dependent on the initial concentration and growth rate of bacteria in the sample [25]. This approach might make pre-incubation unnecessary because tests can be run on a single bacterium. It was also reported that this method avoids MIC measurements because it is not based on standard methodology.

Table 1 also shows that various materials and microfabrication processes can be used to create a lab on a chip during the study of *S. aureus*. As a result, silicon wafers can be produced with nanometer-scale chip features using photolithography. In microfluidic chips, common compartment types include reservoirs, chambers, and microchannels. Additionally, functional components such as valves, mixers, and pumps are made to transfer liquid in a particular manner. According to the findings of this review, polydimethylsiloxane silicone rubber is largely used in lab settings for the creation of the lab on a chip.

Different strains of *S. aureus* were explored in the same trial. Moreover, most of the experiments were performed considering the recommendations of CLSI [39] and EUCAST [40] with minor modifications.

The susceptibility of *S. aureus* to the following antibiotics was evaluated: gentamicin, ciprofloxacin, moxifloxacin, tobramycin, ceftazidime, meropenem, vancomycin, linezolid, chloramphenicol, flucloxacillin, ampicillin, spectinomycin, streptomycin, amikacin, norfloxacin, ofloxacin, tetracycline, oxacillin, ampicillin, cephalosporin, cephalotin, cefoxitin, levofloxacin, and erythromycin. Most studies also considered MIC ranges according to CLSI [39] and EUCAST [40] and evaluated the lab on a chip on several antibiotics at the same time. It is also important to note that microfluidics works with volumes in the microliter range, whereas standard approaches work with milliliter volumes.

As was described here, lab-on-a-chip devices for assessing antimicrobial susceptibility often utilize various types of sensors to detect and measure the response of microorganisms to antimicrobial agents. These sensors are integrated into the microfluidic platforms to provide rapid and accurate analysis of antimicrobial susceptibility. Some common types of sensors used in lab on a chip system for antimicrobial susceptibility testing include microfluidic optical sensors, microfluidic impedance sensors, microfluidic electrochemical sensors, microfluidic biosensors, microfluidic piezoelectric sensors, and microfluidic acoustic sensors.

The level of risk of bias was minimal across all experiments examined (Table 2). However, the aims, conception, and methods of each model varied. For these reasons, a quantitative assessment is challenging.

## 4. Discussion

**Figure 2:**
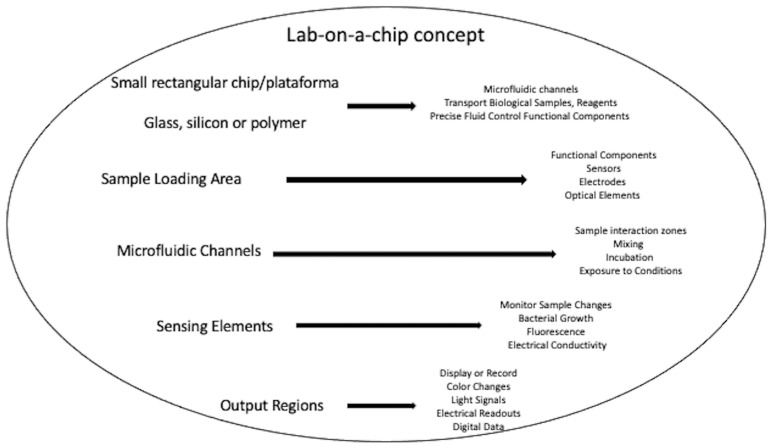
Lab-on-a-chip concept.

An illustrative depiction of the lab-on-a-chip concept includes a small, rectangular chip or substrate that serves as a platform for miniaturized laboratory functions (Figure 2). The chip is typically made of materials such as glass, silicon, or polymer, and it contains a network of microfluidic channels, chambers, and functional components. On the chip, it can see a variety of tiny channels interconnected like a maze. These channels are designed to transport fluids, such as biological samples, reagents, or cell cultures. The microfluidic channels allow precise control over the flow of fluids, enabling various processes. Along the channels, it can visualize functional components, such as sensors, electrodes, and optical elements. These components are integrated directly into the chip and can measure parameters like pH, temperature, fluorescence, or electrical signals. In one part of the chip, there is a sample loading area, where a small volume of the biological sample is introduced into the system. The sample may contain bacteria, cells, or molecules that need to be analyzed or tested. By following the channels, multiple regions where the sample interacts with different reagents or undergoes various processes are noticed. For example, one region might be designated for mixing the sample with specific antibiotics or other test compounds. Further along, areas are seen where the sample is exposed to different conditions, such as varying temperatures or incubation times. These conditions mimic specific aspects of a laboratory environment but at a much smaller scale. In another section of the chip, there are sensing elements. These sensors can monitor changes in the sample’s properties, such as bacterial growth, fluorescence, or electrical conductivity. The sensors provide real-time data, allowing researchers to observe how the sample responds to different treatments. The final part of the chip contains output regions, where the results of the analysis or testing are displayed or recorded. This could be in the form of color changes, light signals, electrical readouts, or digital data.

Growth techniques for microorganisms, including *S. aureus*, for in vitro phenotypic testing have often lagged behind other technological advances. Even currently, with remarkable automation and robotics, the well plate is still referred to as the ultimate high-throughput platform [27]. Because of the complexities of the procedures involved in the assay, it is frequently limited to the use of 96-well plates when culturing microbial biofilms. Even though liquid-handling stations can help with the initial dispensing of cells into wells, eliminating the need for human interaction to complete the rest of the experiment is difficult [26,27]. The delicate nature of biofilms, the three-dimensional architecture, various morphologies, possible disturbance, and cell loss during the washing processes required in the test are some of the problems that impede the full automation of these assays [27,28,29]. To address all these challenges, a completely automated lab-on-a-chip platform for biofilm and planktonic culturing in nanoscale volumes has been developed. Antimicrobial susceptibility testing and ultra-high-throughput screening applications, such as those meant to prevent biofilm formation and those designed to assess established biofilms, are excellent for lab on a chip [25,26,27].

As noted in this systematic review, antimicrobial susceptibility testing of *S. aureus* is a clinically important application of lab-on-a-chi- in antibiotics research [17]. Slow-growing bacteria may take more than a day to be identified in phenotypic antimicrobial susceptibility testing because they must divide enough times to be seen under a microscope [17,19,25,26,27]. This review demonstrated that there are several methods to solve the detection time problem in antimicrobial susceptibility testing of *S. aureus* using a lab on a chip; however, this approach also has its challenges. 

It was observed here that following the growth of *S. aureus* in the presence of antibiotics is a relevant technique [25,26,27,28,29,30,31,32,33,34,35,36,37,38]. Such assays have been found to produce MIC quickly [26,29,30,31,33]. Nevertheless, methodologies created on single-cell imaging necessitate high-resolution optics and time-lapse imaging at many locations [17]. This review also found that there are different ways to perform rapid microfluidic antimicrobial susceptibility testing of *S. aureus*. Although most of the studies included in this systematic review reported rapid assessments of up to two hours, shorter times of up to 10–30 min have also been described [25,33]. Solely microfluidic phenomolecular tests have been presented to produce antimicrobial susceptibility testing findings from clinical specimens in such a brief period; however, as was observed here, optical- or sensor-based microfluidic approaches have also shown effective results in a very short time [17].

Integrating various experimental processes into a single platform can also reduce the number of modifications performed by researchers, hence the danger of contamination and methodological unpredictability [14,41]. As was observed here, droplet-based devices are an excellent example of the integrative power of microfluidics [26]. This time savings and low technical manipulation are unquestionably essential considerations for the usage of microfluidic devices in a clinical setting, where correct management of infected patients with *S. aureus* demands predictable and rapid findings [14].

As was observed in this systematic review, it is also important to highlight that whereas conventional approaches are limited to performing analysis on flat 2D surfaces, including culture flasks, Petri dishes, or well plates, lab on a chip proposes a novel range of constituents with interesting features, the most notable of which being polydimethylsiloxane [14]. It is good for cellular and microbiological cultures due to its biocompatibility and gas permeability, but its optical transparency also enables microscope viewing and analysis. It is important to note that its main benefit is the ability to combine it with various materials, resulting in significant equipment composition diversity. Therefore, microfluidics allows for the application of diverse stimuli, such as fluid flow or tactic gradients, in heterogeneous or even three-dimensional settings [14,17,25,26,27,28,30,31,32,33].

As was described in this systematic review, the disparity in volume and *S. aureus* populations employed in microfluidics versus traditional approaches is also an important factor to recognize [14]. Microfluidics works with volumes in the microliter range, whereas standard approaches work with milliliter volumes. This minor volume transforms microfluidic strategies into portable implements, allowing for cost reductions in reagents, resources, and area, as well as more exact control of the study’s biological and physical parameters [14,26,29,30,31]. The quantity of bacteria in a lab on a chip is reduced to hundreds or even solitary bacterium, resulting in more exact results on a single-cell scale [14].

As was described here, various types of sensors were described in this systematic review. The choice of the sensor depends on the specific requirements of the antimicrobial susceptibility assay, such as sensitivity, detection limit, and compatibility with the microfluidic platform. As was observed here, the integration of these sensors into lab-on-a-chip systems enables rapid, automated, and miniaturized antimicrobial susceptibility testing, providing valuable tools for clinical diagnostics, research, and antimicrobial stewardship [41,42].

Lab-on-a-chip technology, Raman spectroscopy, and Matrix-Assisted Laser Desorption/Ionization Time-of-Flight Mass Spectrometry (MALDI-TOF MS) are all powerful tools with distinct capabilities for antibiotic susceptibility testing. Lab-on-a-chip technology excels in providing real-time, miniaturized, and integrated platforms for phenotypic and genotypic antibiotic susceptibility testing [41,42]. Raman spectroscopy offers label-free, non-destructive analysis and the ability to study subtle cellular changes related to antibiotic response, while MALDI-TOF MS provides high-throughput bacterial identification and potential insights into protein expression changes linked to antibiotic resistance. Each technology offers unique strengths for antibiotic susceptibility testing, and their potential varies depending on the specific application and research objectives [43]. Integrating these techniques and combining their capabilities may lead to comprehensive and accurate assessments of antimicrobial susceptibility in the future.

Lab-on-a-chip systems designed for assessing antimicrobial susceptibility typically require aseptic environments to ensure accurate and reliable results. The reason for this is that antimicrobial susceptibility testing involves evaluating the response of microorganisms to antibiotics, and any contamination from external sources could interfere with the test results and lead to misleading interpretations [41,42,43].

Although the great advantages of lab on a chip are evident, this alternative also presents some limitations. Despite the various benefits indicated for polydimethylsiloxane, several disadvantages have been reported. Its main restriction in the cellular sector is its ability to absorb tiny hydrophobic molecules or biomolecules, such as proteins, which can interfere with assay results [14,41]. Therefore, to avoid these events, methods that change the surface of polydimethylsiloxane are being established [42]. On the other hand, there is a clear lack of established protocols in a lab on a chip-based investigation [44]. Microorganisms such as *S. aureus* are mostly observed through microscopy techniques, which necessitate the use of reporter-labeled bacteria and/or specialized microscope equipment [14,45]. This limits lab-on-a-chip experiments to visual inspection and model microorganisms, and it is evident that molecular characterization is lacking [14]. However, as was described in this systematic review, the elaboration of droplet-based technologies has resulted in significant progress in this sector, allowing for the study of *S. aureus* at the single-cell level [26]. 

This systematic review has several shortcomings as well. The existing evidence is based on a few in vitro experiments that may have therapeutic applications. Besides, these studies had different objectives, designs, and methodologies, giving them great heterogeneity. The included investigations, on the other hand, exhibited a low degree of risk and have conceivable applicability in antibiotic susceptibility testing.

Antimicrobial susceptibility testing through lab-on-a-chip systems offers several advantages, such as rapid, miniaturized, and automated testing [41,42,43,44]. However, there are also challenges and perspectives that need to be considered for the successful implementation and widespread adoption of this technology. One of the significant challenges is the need for standardization of antimicrobial susceptibility testing protocols on lab-on-a-chip platforms [41,43]. Ensuring consistency in testing methods and interpretation of results is essential for reliable and comparable data across different systems and laboratories. Preparing samples for testing on a lab-on-a-chip system can be challenging, especially when dealing with complex clinical samples containing multiple pathogens or heterogeneous populations of microorganisms. Sample preparation techniques must be optimized to ensure accurate results [41,44]. Effective antimicrobial susceptibility testing requires the integration of multiple parameters, such as bacterial growth, cell viability, and specific markers of antibiotic resistance. Integrating different sensors and analytical techniques into a single chip while maintaining sensitivity and specificity can be demanding. To address the growing problem of multi-drug resistance, lab-on-a-chip systems need to incorporate multiplexing capabilities to assess the susceptibility of multiple antibiotics simultaneously [41,42]. Developing multiplexed assays on a small chip can be technically challenging. Handling the vast amount of data generated by lab-on-a-chip systems can be overwhelming. The development of automated data analysis algorithms and user-friendly interfaces is crucial to ensure accessible and actionable results [41,42,43,44]. In short, lab-on-a-chip technology offers numerous advantages, especially in terms of miniaturization, speed, and integration of lab processes. However, it also comes with challenges related to fabrication complexity, material compatibility, and scalability [46,47,48]. The choice to implement lab-on-a-chip technology should be based on the specific needs and constraints of the application or research project.

### Future Perspective

The future perspective of lab-on-a-chip technology in the clinical field is promising, and it holds the potential to transform various aspects of healthcare and diagnostics:

1. Point-of-Care Diagnostics: lab-on-a-chip devices are likely to become integral for point-of-care diagnostics. They can enable rapid and accurate testing at or near the patient’s location, reducing the need for centralized laboratories and providing quicker results for timely decision-making in emergency situations and routine healthcare [47].
2. Personalized Medicine: lab-on-a-chip technology could play a crucial role in personalized medicine by allowing for the rapid and cost-effective analysis of an individual’s genetic makeup, enabling tailored treatment plans and medication choices based on a patient’s unique genetic profile [49].
3. Early Disease Detection: The sensitivity and specificity of lab-on-a-chip devices make them ideal for early disease detection. They can detect biomarkers associated with diseases like cancer, diabetes, and infectious diseases at their earliest stages, increasing the chances of successful treatment and reducing healthcare costs [47].
4. Remote Monitoring: Miniaturized sensors and microfluidic devices can be integrated into wearable or implantable devices, facilitating continuous monitoring of patients’ health parameters. This real-time data can be sent to healthcare providers, enabling proactive interventions and better management of chronic conditions [50].
5. Drug Development: lab-on-a-chip technology can streamline drug development processes by allowing for high-throughput screening of potential drug candidates and the study of their effects on cells or tissues. This can accelerate the development of new drugs and therapies [51].
6. Telemedicine and Telehealth: The portability of lab-on-a-chip devices makes them suitable for telemedicine and remote health consultations. Patients in remote or underserved areas can access high-quality diagnostics and medical advice through telehealth platforms [43,47].
7. Reduced Healthcare Costs: By enabling early diagnosis, targeted treatments, and efficient monitoring, lab-on-a-chip technology can potentially reduce overall healthcare costs by preventing expensive late-stage interventions and hospitalizations [43,48].
8. Global Health Impact: In resource-limited settings and developing countries, lab-on-a-chip devices can provide affordable and accessible diagnostic tools for diseases, helping to address global health challenges [52].
9. Advanced Imaging and Analysis: Future lab-on-a-chip systems may incorporate advanced imaging and analysis techniques, such as artificial intelligence and machine learning, to enhance the accuracy and speed of diagnoses and data interpretation [53].

Research in the clinical assessment of *S. aureus* susceptibility using lab-on-a-chip technology has the potential to significantly impact the diagnosis and treatment of *S. aureus* infections. As observed in the current systematic review, lab-on-a-chip technology can be used to perform rapid AST for *S. aureus* isolates. Traditional AST methods can take 24–48 h [13,14,15], delaying the initiation of appropriate antibiotic therapy. Lab-on-a-chip can provide results within hours, allowing healthcare providers to select the most effective antibiotic sooner and reducing the risk of treatment failure and the spread of antibiotic-resistant strains. In addition to these promising results, lab-on-a-chip has other applications in clinical research on *S. aureus*. Lab-on-a-chip can be designed to identify the specific strain or type of *S. aureus*, including MRSA. Rapid and accurate identification is crucial for selecting the appropriate antibiotics, as MRSA is often resistant to common antibiotics [28]. Lab-on-a-chip technology can be used in surveillance efforts to monitor antibiotic resistance trends in *S. aureus* [54]. This data can help healthcare institutions and public health agencies track the prevalence of antibiotic-resistant strains, guiding antibiotic stewardship programs and infection control measures. Miniaturized lab-on-a-chip devices can also be used at the point of care, such as in clinics or emergency departments, to quickly assess *S. aureus* susceptibility [55]. This can lead to more targeted and effective treatment decisions, especially in cases of severe infections. Moreover, by rapidly determining the susceptibility of *S. aureus* to various antibiotics, lab-on-a-chip technology can support personalized treatment plans for patients [49]. This can optimize antibiotic selection and dosing, reducing the risk of adverse effects and improving patient outcomes. Lab-on-a-chip technology can aid in the development of novel antibiotics and therapies for *S. aureus* infections [27]. Researchers can use microfluidic devices to test the efficacy of new drug candidates and study the mechanisms of antibiotic resistance. In summary, research in the clinical assessment of *S. aureus* susceptibility using lab-on-a-chip technology has practical applications that can enhance the diagnosis and treatment of *S. aureus* infections, reduce antibiotic resistance, and improve patient care in various healthcare settings. It represents a promising approach to addressing the challenges posed by *S. aureus* and antibiotic resistance in clinical practice.

## 5. Conclusions

The potential use of lab-on-a-chip technology for assessing the antibiotic susceptibility of *S. aureus* is highlighted by its capability to quickly provide essential minimum inhibitory concentration data. This data can greatly assist in the selection of the most appropriate antibiotics and their dosages for addressing complex infections caused by this microorganism. Furthermore, the swift antibiotic susceptibility testing made possible by lab-on-a-chip holds great potential for improving the suitability and effectiveness of treatment in clinical environments. It increases efficiency and conserves both time and resources.

## Figures and Tables

**Table 1:** Principal characteristics of the included studies.

Authors, Publication Date	Materials and Sensors Used	Culture and Growing Conditions	Antibiotics and MIC	Main Results
Lee et al. 2021 [25]	The chip consisted of two polydimethylsiloxane layers (air channel and liquid channel layers) and a glass substrate.	Methicillin-resistant *S. aureus* was inoculated and adjusted to ~10^8^ colony-forming units/mL with saline solution. The bacteria suspension was then fine-tuned to the tested concentration in a colorimetric media containing brain heart infusion broth supplemented with an oxidation/reduction dye.	Antibiotics: Ceftazidime, meropenem, vancomycin, gentamicin, and linezolid. MIC: The MIC values of the tested antibiotics were determined by standard broth microdilution procedures as described in the Clinical & Laboratory Standards Institute guidelines.	Time spent: The entire liquid operation, including uniform sample distribution, antibiotics of 2-fold serial dilutions, and multiple-drug combinations for on-chip antimicrobial susceptibility tests, could be automatically completed within 10 min.
Spencer et al. 2020 [33]	Photolithography and wafer bonding were used to create microfluidic chips.	Clinical breakpoints were based on epidemiological cut-off values extracted from bacterial culture collections and established antibiotic concentrations that allow MIC test results to be used to identify bacterial isolates (EUCAST v10).	Antibiotic: Cefoxitin MIC: The MIC of an antibiotic was calculated by measuring the electrical response of the same isolate when exposed to varied antibiotic concentrations. The electrical MIC calculated using iFAST was compared to the MIC calculated with traditional broth micro-dilution.	Time spent: In a 30-min incubation window, the recorded electrical features reflect the phenotypic response of the bacteria to the method of action of a certain antibiotic.
Wistrand-Yuen et al. 2020 [32]	A microfluidic chip was created using three-dimensional (3D) printed molds for polydimethylsiloxane casting and a 3D printed chip holder with an integrated reservoir cover.	The EUCAST Development Laboratory provided all bacterial strains. *S. aureus* isolates (n = 10) with varying degrees of sensitivity to the drugs tested were utilized. Cation-adjusted Müller-Hinton broth and MH-II agar were used to culture all strains.	Antibiotics: Gentamicin, ofloxacin, and tetracycline. MIC: According to the EUCAST, the antibiotic doses employed in the studies in the source solutions for gradient creation were 2.5-fold greater than the clinical breakpoints for resistance for the tested antibiotic against the tested specimen in each case.	Time spent: Within 2 to 4 h, stable MIC values were achieved, and the results indicated categorical agreement with reference MIC values established by broth microdilution in 86% of the instances.
Huang et al. 2020 [26]	Both the microwell and microchannel devices were made with the help of the soft lithography technique.	*S. aureus* was transfected with the green fluorescent protein for fluorescent imaging to verify the effectiveness of the bacterium trapping. Bacteria were first re-solubilized in 5 mL of Mueller Hinton Broth and cultured in an orbital shaking incubator.	Antibiotic: Chloramphenicol. MIC: The study from the Clinical & Laboratory Standards Institute states that the chosen antibiotic concentration was the required concentration to identify resistant bacteria.	Time spent: the Microwell-surface-enhanced Raman scattering system demonstrated a 2-h antibiotic susceptibility test on susceptible and resistant *S. aureus*.
Lee et al. 2019 [19]	Polymethylmethacrylate molds of an air channel layer and a liquid channel layer were microfabricated by a computer-numerical-control engraving machine.	The isolated bacteria were inoculated in saline solution for the antimicrobial susceptibility testing assay and then adjusted with a nephelometer to 0.5 McFarland turbidity standards. The bacteria suspension was then diluted to 10^6^ CFU mL^−1^ with the pH-dependent colorimetric broth.	Antibiotics: Vancomycin, gentamicin, and linezolid. MIC: Tests with methicillin-resistant *S. aureus* were conducted using vancomycin and linezolid, whereas Gram-negative bacteria were treated with gentamicin. The tested antibiotics’ MIC values were calculated using conventional broth dilution techniques in accordance with the Clinical & Laboratory Standards Institute recommendations (CLSI).	Time spent: The ability of this integrated microfluidic system to not only provide MIC values for two antibiotics within the same incubation time (24 h) but also increase seven reaction chambers (from 5 to 12) on a single chip may improve the throughput for antimicrobial susceptibility testing of a single drug and the operational flexibility for clinicians.
Yi et al. 2019 [34]	The integrated dielectrophoresis (DEP)-antimicrobial susceptibility testing SlipChip device was created.	*S. aureus* ATCC 6538P without exogenous plasmid was employed as the bacterial strain. Single colonies of these strains were injected into 5 mL of Mueller-Hinton broth (CAMHB) for overnight culture at 37 °C from cation-adjusted CAMHB agar plates.	Antibiotics: ampicillin, moxifloxacin, cephalothin, and meropenem. MIC: The entropy-based image analysis method was used to estimate bacterial antimicrobial susceptibility. The bright-field images were separated into matrices of 20 20-pixel segments, which correspond to the size of a few bacterial cells.	Time spent: After a positive blood culture, this inexpensive gadget can provide reliable antimicrobial susceptibility testing values to clinicians within 3–8 h, allowing for early antimicrobial medication administration.
Sun et al. 2019 [35]	3-D-shaped channel design built with the help of computer simulation.	*S. aureus* (ATCC 29213) was tested on the polypropylene chip, and the results were compared to testing on the polydimethylsiloxane chip.	Antibiotics: Ampicillin, gentamicin, tetracycline, and erythromycin. MIC: The MIC values of the various antibiotics were determined using CLSIS data.	Time spent: This technology enables the reliable tracking of individual cells and the acquisition of antimicrobial susceptibility testing data in 1–3 h.
Kang et al. 2019 [36]	Photolithography of the SU-8 master template, soft lithography of the polydimethylsiloxane replicas, and plasma bonding of the device to microscope slides.	Trypticase Soy Agar/Broth was used to cultivate *S. aureus* (ATCC 29213). The bacteria samples were prepared according to ATCC methods. Concentration of 20% glycerol stock. Stocks were kept at −80 °C.	Antibiotics: oxacillin and tetracycline. MIC: The MIC of antibiotics in droplets was calculated when there was a low or no increase in bacterial quantity in droplets and a significant delay in the bacteria’s average doubling time to 2 h or more.	Time spent: This method is substantially faster than typical antimicrobial susceptibility testing (30 min versus 16–24 h).
Hou et al. 2017 [37]	The device’s fundamental structure consists of a chamber and two channels that link to both sides of it.	*S. aureus* SH1000 was isolated and cultivated for 18 h in 5 mL of LB broth. Mueller-Hinton broth medium containing approximately 1.0 108 CFU/mL of each type of bacterium strain was combined with 1% low melting temperature agarose.	Antibiotic: Vancomycin. MIC: The MIC was calculated using 90% growth inhibition (on a grayscale, 75 and larger). As reference studies, CLSI Protocol MIC determination and Etest were performed.	Time spent: The chip method saves a significant amount of time and labor because experiments can be completed in 4 h, and findings may be examined in real-time.
Srinivasan et al. 2017 [27]	The nBioChip is printed robotically, handled carefully, then scanned with a typical microarray reader.	*S. aureus* strain UAMS 1 frozen stock was subcultured onto tryptic soy agar plates and propagated in 10 mL of tryptic soy broth at 37 °C in an orbital shaker. A 100-μL volume of the overnight liquid culture was subcultured into 10 mL of tryptic soy broth for 3 h to capture cells in the log phase.	Antibiotics: Vancomycin, clindamycin, ciprofloxacin, tobramycin sulfate, methicillin, and linezolid. MIC: The antibiotics were all diluted to a maximum concentration of 100 g/mL in phosphate-buffered saline. The antimicrobial susceptibility assays required additional dilutions to be done in phosphate-buffered saline.	Time spent: It was shown that the susceptibility profiles for the four medications were noticeably different at 6-, 12-, 18-, or 24-h following exposure. In contrast to vancomycin, which had a much steeper response curve, the biofilms responded to ciprofloxacin, clindamycin, and linezolid more gradually.
Malmberg et al. 2016 [38]	CellDirector 3D is a cell-based assay that employs microfluidics to produce a linear gradient of chemicals via diffusion through a microscale test chamber.	*S. aureus* (vancomycin susceptible), *S. aureus* ATCC 29213, and heteroresistant vancomycin-intermediate *S. aureus* ATCC 700698 were used. Before the trials, all strains were kept at −80 °C and streaked on Mueller-Hinton II blood agar plates.	Antibiotic: Vancomycin. MIC: The CellDirector 3D test was initially evaluated using *S. aureus* vancomycin susceptible and heteroresistant vancomycin-intermediate *S. aureus*, defined as MIC 2 mg/L but with a subpopulation with MIC > 2 mg/L). Etest’s MIC determination was carried out in accordance with the manufacturer’s instructions.	Time spent: The time to probable readout was between 2 and 5 h. The microfluidic assay under consideration has the potential to give rapid and accurate MICs utilizing samples from positive clinical blood cultures.
Abeyrathne et al. 2016 [28]	Laser ablation lithography was used to create and design interdigitated electrodes.	Reference strains of *S. aureus* that were both methicillin-susceptible (ATCC 25923) and resistant (ATCC 33591) were used. The bacterial strains were grown for 18 h at 37 °C in Luria broth.	Antibiotics: Flucloxacillin MIC: In Luria broth media, different flucloxacillin concentrations were created. In a closed petri dish, 20 μL of the antibiotic solution was put on filter papers that were 1 mm × 1 mm in size.	Time spent: A lab on a chip demonstrated the ability to detect *S. aureus* in a sample in under an hour.
Hou et al. 2014 [29]	A 3D microfluidic culture system was placed above an inverted optical microscope equipped with a phase contrast condenser.	*S. aureus* SH1000 bacteria were the strains used. From Lysogeny broth agar plates, single colonies were selected and cultivated in 5 mL of Lysogeny broth medium for 5–6 h, or until the OD_595_ reached 0.3–0.4.	Antibiotics: Ampicillin, spectinomycin, tetracycline, and vancomycin. MIC: The MIC was determined by the Clinical & Laboratory Standards Institute protocol and by the Etest in accordance with the supplier’s instructions to compare this on-chip test with conventional static techniques.	Time spent: The entire procedure, including data analysis, took 2.5–4 h. High-resolution growth curves were produced from the same analysis.
Choi et al. 2013 [30]	Polydimethylsiloxane was used to create the microfluidic agarose channel chip, and polydimethylsiloxane-coated glass was used to assemble it.	A common Clinical & Laboratory Standards Institute bacteria (*S. aureus* ATCC 29213) was combined with agarose to validate the microfluidic agarose channel technology. The bacteria were subcultured in a new cation-adjusted Mueller-Hinton broth medium for one hour.	Antibiotics: Amikacin, norfloxacin, gentamicin, and tetracycline. MIC: According to the MIC range of the Clinical & Laboratory Standards Institute, six channels were chosen to test six different antibiotic concentrations.	Time spent: The full antimicrobial susceptibility test procedure lasts 3–4 h, and the results are equally accurate to the Clinical & Laboratory Standards Institute’s traditional antimicrobial susceptibility test results.
Kalashnikov et al. 2012 [31]	A polydimethylsiloxane layer was placed on top of an epoxy-coated glass slide to construct the microfluidic channels.	Methicillin-resistant or methicillin-susceptible clinically relevant *S. aureus* strains were used. *S. aureus* strains ST22, ST80, SF8300, TCH959, and other meticillin-resistant *S. aureus* strains were investigated.	Antibiotics: methicillin and oxacillin. MIC: The *S. aureus* MIC value of 60 ng/mL, obtained from the prototype strains using conventional microdilution broth methods, was almost 100 times lower than this lysostaphin concentration. Rates of killing for the bacterial samples in the presence and absence of oxacillin were assessed.	Time spent: A metric was developed to distinguish between susceptible and resistant staphylococci based on normalized fluorescence levels following 60 min of exposure to stress and antibiotics using model susceptible and *resistant S. aureus* strains.
Boedicker et al. 2008 [20]	Devices made of polydimethylsiloxane with channel widths between 200 and 800 μm were created.	*S. aureus* ATCC# 25923 and *S. aureus* ATCC# 43300 cells were obtained from ATCC. Using Miller’s Luria-Bertani medium formulation, which contains 30% glycerol, stock solutions of the cells were created and kept at −80 °C.	Antibiotics: Ampicillin, oxacillin, cephalosporin, cefoxitin, levofloxacin, vancomycin, and erythromycin. MIC: According to the British Society for Antimicrobial Chemotherapy procedures for antimicrobial susceptibility testing, antibiotics were tested at the breakpoint concentration.	Time spent: In less than 7 h, a bacterial sample’s thorough functional characterization was completed. Additionally, it was shown that a bacterium in a 1 nL plug could be found in as short as 2 h. Volume after a single bacterium was contained and examined in plugs.

**Table 2:** Risk of bias of the assessed investigations.

Study	* Design	** Measures	*** Analysis Statistical	Clinical Significance	Total
Lee et al. 2021 [25]	4	3	4	1	12
Spencer et al. 2020 [33]	4	3	4	1	12
Wistrand-Yuen et al. 2020 [32]	4	3	4	1	12
Huang et al. 2020 [26]	4	3	4	1	12
Lee et al. 2019 [19]	4	3	4	1	12
Yi et al. 2019 [34]	4	3	4	1	12
Sun et al. 2019 [35]	4	3	4	1	12
Kang et al. 2019 [36]	4	3	4	1	12
Hou et al. 2017 [37]	4	3	4	1	12
Srinivasan et al. 2017 [27]	4	3	4	1	12
Malmberg et al. 2016 [38]	4	3	4	1	12
Abeyrathne et al. 2016 [28]	4	3	4	1	12
Hou et al. 2014 [29]	4	3	4	1	12
Choi et al. 2013 [30]	4	3	4	1	12
Kalashnikov et al. 2012 [31]	4	4	5	1	14
Boedicker et al. 2008 [20]	4	3	4	1	12

* Design includes aim, sample, baseline features and co-interventions. ** Measures contain measurement mode, blinding clinician and statistician, explained consistency, and level of agreement. *** Statistical analysis involves suitable analysis, co-interventions, subgroup analysis, statistical significance, and confidence intervals.

## Data Availability

The data obtained in this review were pooled from the included investigations.

## References

[1] Aslam B., Khurshid M., Arshad M.I., Muzammil S., Rasool M., Yasmeen N., Shah T., Chaudhry T.H., Rasool M.H., Shahid A. (2021). Antibiotic Resistance: One Health One World Outlook. Front. Cell. Infect. Microbiol..

[2] Ferri M., Ranucci E., Romagnoli P., Giaccone V. (2017). Antimicrobial resistance: A global emerging threat to public health systems. Crit. Rev. Food Sci. Nutr..

[3] Uddin T.M., Chakraborty A.J., Khusro A., Zidan B.R.M., Mitra S., Bin Emran T., Dhama K., Ripon K.H., Gajdács M., Sahibzada M.U.K. (2021). Antibiotic resistance in microbes: History, mechanisms, therapeutic strategies and future prospects. J. Infect. Public Health.

[4] Hernando-Amado S., Coque T.M., Baquero F., Martínez J.L. (2019). Defining and combating antibiotic resistance from One Health and Global Health perspectives. Nat. Microbiol..

[5] Tacconelli E., Carrara E., Savoldi A., Harbarth S., Mendelson M., Monnet D.L., Pulcini C., Kahlmeter G., Kluytmans J., Carmeli Y. (2018). WHO Pathogens Priority List Working Group. Discovery, research, and development of new antibiotics: The WHO priority list of antibiotic-resistant bacteria and tuberculosis. Lancet Infect. Dis..

[6] Guo Y., Song G., Sun M., Wang J., Wang Y. (2020). Prevalence and Therapies of Antibiotic-Resistance in *Staphylococcus aureus*. Front. Cell. Infect. Microbiol..

[7] Silva-de-Jesus A.C., Ferrari R.G., Panzenhagen P., Conte-Junior C.A. (2022). *Staphylococcus aureus* biofilm: The role in disseminating antimicrobial resistance over the meat chain. Microbiology.

[8] Lewis N., Leaptrot D., Witt E., Smith H., Hebden J.N., Wright M.O. (2023). Health Care-Associated Infections Studies Project: An American Journal of Infection Control and National Healthcare Safety Network Data Quality Collaboration Case Study—Laboratory-Identified Event Reporting Validation. Am. J. Infect Control..

[9] Sydnor E.R., Perl T.M. (2011). Hospital epidemiology and infection control in acute-care settings. Clin. Microbiol. Rev..

[10] Leekha S., Terrell C.L., Edson R.S. (2011). General principles of antimicrobial therapy. Mayo Clin. Proc..

[11] Braykov N.P., Morgan D.J., Schweizer M.L., Uslan D.Z., Kelesidis T., A Weisenberg S., Johannsson B., Young H., Cantey J., Srinivasan A. (2014). Assessment of empirical antibiotic therapy optimisation in six hospitals: An observational cohort study. Lancet Infect. Dis..

[12] Renner L.D., Zan J., Hu L.I., Martinez M., Resto P.J., Siegel A.C., Torres C., Hall S.B., Slezak T.R., Nguyen T.H. (2017). Detection of ESKAPE Bacterial Pathogens at the Point of Care Using Isothermal DNA-Based Assays in a Portable Degas-Actuated Microfluidic Diagnostic Assay Platform. Appl. Environ. Microbiol..

[13] Balouiri M., Sadiki M., Ibnsouda S.K. (2016). Methods for in vitro evaluating antimicrobial activity: A review. J. Pharm. Anal..

[14] Pérez-Rodríguez S., García-Aznar J.M., Gonzalo-Asensio J. (2022). Microfluidic devices for studying bacterial taxis, drug testing and biofilm formation. Microb. Biotechnol..

[15] Bodor A., Bounedjoum N., Vincze G.E., Kis E., Laczi K., Bende G., Szilágyi Á., Kovács T., Perei K., Rákhely G. (2020). Challenges of unculturable bacteria: Environmental perspectives. Rev. Environ. Sci. Biotechnol..

[16] Lebeaux D., Chauhan A., Rendueles O., Beloin C. (2013). From in vitro to in vivo Models of Bacterial Biofilm-Related Infections. Pathogens.

[17] Postek W., Pacocha N., Garstecki P. (2022). Microfluidics for antibiotic susceptibility testing. Lab. Chip..

[18] Kaprou G.D., Bergšpica I., Alexa E.A., Alvarez-Ordóñez A., Prieto M. (2021). Rapid Methods for Antimicrobial Resistance Diagnostics. Antibiotics.

[19] Lee W.B., Chien C.C., You H.L., Kuo F.C., Lee M.S., Lee G.B. (2019). An integrated microfluidic system for antimicrobial susceptibility testing with antibiotic combination. Lab Chip.

[20] Boedicker J.Q., Li L., Kline T.R., Ismagilov R.F. (2008). Detecting bacteria and determining their susceptibility to antibiotics by stochastic confinement in nanoliter droplets using plug-based microfluidics. Lab Chip.

[21] Hou H.W., Bhattacharyya R.P., Hung D.T., Han J. (2015). Direct detection and drug-resistance profiling of bacteremias using inertial microfluidics. Lab Chip.

[22] Sun H., Liu Z., Hu C., Ren K. (2016). Cell-on-hydrogel platform made of agar and alginate for rapid, low-cost, multidimensional test of antimicrobial susceptibility. Lab Chip.

[23] Moher D., Liberati A., Tetzlaff J., Altman D.G., PRISMA Group (2009). Preferred reporting items for systematic reviews and meta-analyses: The PRISMA Statement. Open Med..

[24] Ehsani S., Mandich M.A., El-Bialy T.H., Flores-Mir C. (2009). Frictional resistance in self-ligating orthodontic brackets and conventionally ligated brackets: A systematic review. Angle Orthod..

[25] Lee W.B., Chien C.C., You H.L., Kuo F.C., Lee M.S., Lee G.B. (2021). Rapid antimicrobial susceptibility tests on an integrated microfluidic device for precision medicine of antibiotics. Biosens. Bioelectron..

[26] Huang H.-K., Cheng H.-W., Liao C.-C., Lin S.-J., Chen Y.-Z., Wang J.-K., Wang Y.-L., Huang N.-T. (2020). Bacteria encapsulation and rapid antibiotic susceptibility test using a microfluidic microwell device integrating surface-enhanced Raman scattering. Lab Chip.

[27] Srinivasan A., Torres N.S., Leung K.P., Lopez-Ribot J.L., Ramasubramanian A.K. (2017). *nBio*Chip, a Lab-on-a-Chip Platform of Mono- and Polymicrobial Biofilms for High-Throughput Downstream Applications. mSphere.

[28] Abeyrathne C.D., Huynh D.H., Mcintire T.W., Nguyen T.C., Nasr B., Zantomio D., Chana G., Abbott I., Choong P., Catton M. (2016). Lab on a chip sensor for rapid detection and antibiotic resistance determination of *Staphylococcus aureus*. Analyst.

[29] Hou Z., An Y., Hjort K., Hjort K., Sandegren L., Wu Z. (2014). Time lapse investigation of antibiotic susceptibility using a microfluidic linear gradient 3D culture device. Lab Chip.

[30] Choi J., Jung Y.-G., Kim J., Kim S., Jung Y., Na H., Kwon S. (2013). Rapid antibiotic susceptibility testing by tracking single cell growth in a microfluidic agarose channel system. Lab Chip.

[31] Kalashnikov M., Lee J.C., Campbell J., Sharon A., Sauer-Budge A.F. (2012). A microfluidic platform for rapid, stress-induced antibiotic susceptibility testing of *Staphylococcus aureus*. Lab Chip.

[32] Wistrand-Yuen P., Malmberg C., Fatsis-Kavalopoulos N., Lübke M., Tängdén T., Kreuger J. (2020). A Multiplex Fluidic Chip for Rapid Phenotypic Antibiotic Susceptibility Testing. mBio.

[33] Spencer D.C., Paton T.F., Mulroney K.T., Inglis T.J.J., Sutton J.M., Morgan H. (2020). A fast impedance-based antimicrobial susceptibility test. Nat. Commun..

[34] Yi Q., Cai D., Xiao M., Nie M., Cui Q., Cheng J., Li C., Feng J., Urban G., Xu Y.C. (2019). Direct antimicrobial susceptibility testing of bloodstream infection on SlipChip. Biosens. Bioelectron..

[35] Sun H., Chan C.W., Wang Y., Yao X., Mu X., Lu X., Zhou J., Cai Z., Ren K. (2019). Reliable and reusable whole polypropylene plastic microfluidic devices for a rapid, low-cost antimicrobial susceptibility test. Lab Chip.

[36] Kang W., Sarkar S., Lin Z.S., McKenney S., Konry T. (2019). Ultrafast Parallelized Microfluidic Platform for Antimicrobial Susceptibility Testing of Gram Positive and Negative Bacteria. Anal. Chem..

[37] Hou Z., An Y., Wu Z. (2017). Dynamic Antibiotic Susceptibility Test via a 3D Microfluidic Culture Device. Methods Mol. Biol..

[38] Malmberg C., Yuen P., Spaak J., Cars O., Tängdén T., Lagerbäck P. (2016). A Novel Microfluidic Assay for Rapid Phenotypic Antibiotic Susceptibility Testing of Bacteria Detected in Clinical Blood Cultures. PLoS ONE.

[39] CLSI (2018). Methods for Dilution Antimicrobial Susceptibility Tests for Bacteria that Grow Aerobically.

[40] Desmet S., Verhaegen J., Glupzcynski Y., Van Eldere J., Melin P., Goossens H., Piérard D., Declercq P., Lagrou K., Boel A. (2016). Development of a national EUCAST challenge panel for antimicrobial susceptibility testing. Clin. Microbiol. Infect..

[41] Halldorsson S., Lucumi E., Gómez-Sjöberg R., Fleming R.M.T. (2015). Advantages and challenges of microfluidic cell culture in polydimethylsiloxane devices. Biosens. Bioelectron..

[42] You J.B., Lee B., Choi Y., Lee C.-S., Peter M., Im S.G., Lee S.S. (2020). Nanoadhesive layer to prevent protein absorption in a poly(dimethylsiloxane) microfluidic device. Biotechniques.

[43] Daniel F., Kesterson D., Lei K., Hord C., Patel A., Kaffenes A., Congivaram H., Prakash S. (2022). Application of Microfluidics for Bacterial Identification. Pharmaceuticals.

[44] Ardila C.M., Jiménez-Arbeláez G.A., Vivares-Builes A.M. (2023). Potential Clinical Application of Organs-on-a-Chip in Periodontal Diseases: A Systematic Review of In Vitro Studies. Dent. J..

[45] Subramanian S., Huiszoon R.C., Chu S., Bentley W.E., Ghodssi R. (2019). Microsystems for biofilm characterization and sensing—A review. Biofilm.

[46] Ezrre S., Reyna M.A., Anguiano C., Avitia R.L., Márquez H. (2022). Lab-on-a-Chip Platforms for Airborne Particulate Matter Applications: A Review of Current Perspectives. Biosensors.

[47] Zhuang J., Yin J., Lv S., Wang B., Mu Y. (2020). Advanced “lab-on-a-chip” to detect viruses—Current challenges and future perspectives. Biosens. Bioelectron..

[48] Cunha M.L., da Silva S.S., Stracke M.C., Zanette D.L., Aoki M.N., Blanes L. (2022). Sample Preparation for Lab-on-a-Chip Systems in Molecular Diagnosis: A Review. Anal. Chem..

[49] Ingber D.E. (2022). Human organs-on-chips for disease modelling, drug development and personalized medicine. Nat. Rev. Genet..

[50] Zhang C., Su Y., Hu S., Jin K., Jie Y., Li W., Nathan A., Ma H. (2020). An Impedance Sensing Platform for Monitoring Heterogeneous Connectivity and Diagnostics in Lab-on-a-Chip Systems. ACS Omega..

[51] Weigl B.H., Bardell R.L., Cabrera C.R. (2003). Lab-on-a-chip for drug development. Adv. Drug. Deliv. Rev..

[52] Gebauer A., Schmidt S., Hoffmann W. (2016). Status and perspective of lab-on-a-chip systems for common diseases: A systematic review from 2003 to 2013. Pers. Med..

[53] Vargas-Ordaz E.J., Gorelick S., York H.M., Liu B., Halls M.L., Arumugam S., Neild A., de Marco A., Cadarso V.J. (2021). Three-dimensional imaging on a chip using optofluidics light-sheet fluorescence microscopy. Lab Chip.

[54] Peter H., Wienke J., Bier F.F. (2017). Lab-on-a-Chip Multiplex Assays. Methods Mol. Biol..

[55] Schulz M., Calabrese S., Hausladen F., Wurm H., Drossart D., Stock K., Sobieraj A.M., Eichenseher F., Loessner M.J., Schmelcher M. (2020). Point-of-care testing system for digital single cell detection of MRSA directly from nasal swabs. Lab Chip.

